# Onion (*Allium cepa* L.) Flavonoid Extract Ameliorates Osteoporosis in Rats Facilitating Osteoblast Proliferation and Differentiation in MG-63 Cells and Inhibiting RANKL-Induced Osteoclastogenesis in RAW 264.7 Cells

**DOI:** 10.3390/ijms25126754

**Published:** 2024-06-19

**Authors:** Danyang Zhang, Xiaoyu Wang, Kezhuo Sun, Jianli Guo, Jia Zhao, Yuesheng Dong, Yongming Bao

**Affiliations:** 1 School of Bioengineering, Dalian University of Technology, Dalian 116024, China; zhangdanyang96@mail.dlut.edu.cn (D.Z.); 32147002@mail.dlut.edu.cn (X.W.); 15204161357@163.com (K.S.); zhaojia01@boan-bio.com (J.Z.); 2 Panjin Institute of Industrial Technology, Dalian University of Technology, Panjin 124221, China; guojianli19880728@163.com; 3 School of Ocean Science and Technology, Dalian University of Technology, Panjin 124221, China

**Keywords:** osteoporosis, *Allium cepa* L., flavonoids, osteoblast, osteoclast

## Abstract

Osteoporosis, a prevalent chronic health issue among the elderly, is a global bone metabolic disease. Flavonoids, natural active compounds widely present in vegetables, fruits, beans, and cereals, have been reported for their anti-osteoporotic properties. Onion is a commonly consumed vegetable rich in flavonoids with diverse pharmacological activities. In this study, the trabecular structure was enhanced and bone mineral density (BMD) exhibited a twofold increase following oral administration of onion flavonoid extract (OFE). The levels of estradiol (E2), calcium (Ca), and phosphorus (P) in serum were significantly increased in ovariectomized (OVX) rats, with effects equal to alendronate sodium (ALN). Alkaline phosphatase (ALP) and tartrate-resistant acid phosphatase (TRAP) levels in rat serum were reduced by 35.7% and 36.9%, respectively, compared to the OVX group. In addition, the effects of OFE on bone health were assessed using human osteoblast-like cells MG-63 and osteoclast precursor RAW 264.7 cells in vitro as well. Proliferation and mineralization of MG-63 cells were promoted by OFE treatment, along with increased ALP activity and mRNA expression of osteoprotegerin (OPG)/receptor activator of nuclear factor-kappaB ligand (RANKL). Additionally, RANKL-induced osteoclastogenesis and osteoclast activity were inhibited by OFE treatment through decreased TRAP activity and down-regulation of mRNA expression-related enzymes in RAW 264.7 cells. Overall findings suggest that OFE holds promise as a natural functional component for alleviating osteoporosis.

## 1. Introduction

Nowadays, osteoporosis is a prevalent bone health issue worldwide that primarily affects the elderly and postmenopausal women [1]. It is characterized by the destruction of bone tissue microstructure, loss of bone mass, and reduced bone strength [2]. In China, the prevalence of osteoporosis in individuals over 40 years old is 5% for men and 20.6% for women [3], placing patients at a high risk of fractures and imposing a significant burden on China’s healthcare system [4].

Bone homeostasis is maintained through continuous bone remodeling, which relies on the balance between osteoclast-mediated bone resorption and osteoblast-driven bone formation [5]. The dysregulation of these dynamic processes ultimately leads to the development of osteoporosis [6]. The osteoprotegerin (OPG)-receptor activator of the nuclear factor-kappaB ligand (RANKL)-receptor activator of the nuclear factor-kappaB (RANK) signaling axis plays a pivotal role in orchestrating the pathogenesis of osteoporosis. Specifically, RANKL, secreted by osteoclasts, stimulates both osteoclast differentiation and subsequent bone resorption upon binding to its cognate receptor RANK expressed on pre-osteoclastic cells’ surfaces [7]. Conversely, OPG, an inhibitory decoy receptor produced by osteoblasts, effectively counteracts RANKL-induced activation of downstream pathways within mature osteoclasts by competitively binding with RANKL itself [8]. Consequently, it is widely acknowledged that maintaining an appropriate ratio between OPG and RANKL serves as a fundamental determinant for regulating physiological bone resorption.

Bisphosphonates, anabolic therapy, and denosumab are commonly employed pharmaceuticals for the treatment of osteoporosis [9]. However, these medications are associated with significant adverse effects such as gastrointestinal damage and an increased risk of osteonecrosis of the jaw [10]. For this reason, it is imperative to explore natural remedies that offer both efficacy and gentleness as alternatives to conventional medicines. In recent years, plant-derived products have emerged as a pivotal approach in the management of osteoporosis [11].

Flavonoids, a group of natural phenolic compounds found in daily consumed vegetables, fruits, and beverages [12], are widely recognized for their diverse pharmacological effects. Notably, flavonoids have been reported to exhibit significant medicinal activity against osteoporosis. Previous studies have demonstrated that genistein can enhance BMD in postmenopausal women with osteopenia [1]. Furthermore, isomeric flavonoids derived from Epimedium exert anti-osteoporotic effects by targeting OPG/RANKL signaling pathways [13]. Onion, known as “the queen of vegetables”, is rich in dietary flavonoids and possesses numerous health benefits, such as antioxidant properties [14] and anti-cancer [15]. Law et al. reported that onion juice could improve antioxidant activities and subsequently improve bone mineral density (BMD) [16]. Despite these efforts, the underlying mechanism responsible for the anti-osteoporotic effect of onions remains largely unexplored.

The primary objective of this study is to investigate the potential of onion flavonoid extract (OFE) as a promising plant-derived nutrient for moderating osteoporosis and elucidate its underlying mechanism, providing a theoretical foundation for the development of functional foods or dietary supplement programs.

## 2. Results

### 2.1. Main Components in the OFE

In the preliminary study, 2.32 g OFE was obtained from 7.50 g dry onion powder. The content of total flavonoids in OFE was determined to be 10.4% using the colorimetry method. To investigate the main components in the OFE, chromatography (HPLC) (Figure S1) and high-resolution liquid chromatography–tandem mass spectrometry (LC−MS/MS) (Table S1) of the four compositions with the top peak areas in HPLC were preliminarily analyzed. Compound 1 was observed at a retention time of 4.9 min in the *m*/*z* 625.1396 mass chromatogram, and its mass spectrum demonstrated a loss of *m*/*z* 162. In addition, the ion at *m*/*z* 463.0863 represented the loss of a single glucose, which would be expected for diglucoside of quercetin [17]. Compound 2 (retention time, RT 6.9 min) with the [M-H]^−^ ion at *m*/*z* 463.0870 was observed to have three major fragments at *m*/*z* 301.0342, 178.9982, and 151.0034. The result suggested that the compound could be appointed as a glycoside of quercetin [18]. What is more, compound 3, at a retention time of 7.3 min, was tentatively in accordance with glucoside of isorhamnetin, which illustrated a satisfactory intensity. It generated a deprotonated molecular ion [M-H]^−^ at *m*/*z* 477.1030, which further gave MS/MS ions at *m*/*z* 315.0495. It was indicated that the molecular formula of compound 4 with the [M-H]^−^ ion at *m*/*z* 301.0347 was C_15_H_10_O_7_, which demonstrated that it might be an aglycon of flavonoid. Its [M-H]^−^ ion generated a characteristic fragment at *m*/*z* 151.0033, owing to the fragmentation of ring-C related to the retro Diels–Alder (RDA) reaction. It was reported that this reaction occurred in flavone aglycone most often. Moreover, the fragmentation of *m*/*z* 273.0393 also occurs in free flavonoids frequently because of the cleaning of CO in ring-C [19]. By comparison of standard substances by HPLC (Figure S2), compound 4 was confirmed as quercetin. The results identified that quercetin and its derivatives and derivatives of isorhamnetin were the main ingredients, in agreement with the studies reported [20]. Quantification by HPLC with quercetin as the equivalent suggested that the content of four major flavonoids (quercetin, its two derivatives, and the derivative of isorhamnetin) of OFE was determined to be 7.4%. Therefore, the total content of quercetin and its derivatives and derivatives of isorhamnetin was 71.9% of total flavonoids.

### 2.2. Effects of OFE on Morphological Parameters by Micro-CT

**Figure 1:**
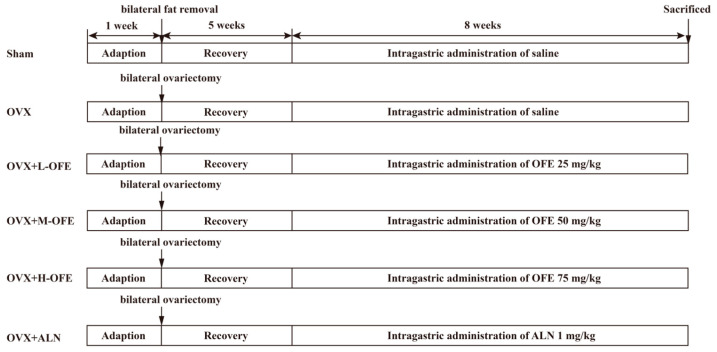
Design of animal experimental study. OVX+L-OFE: OVX rats treated with low-dose OFE, OVX+M-OFE: OVX rats treated with middle-dose OFE, OVX+H-OFE: OVX rats treated with high-dose OFE, OVX+ALN: OVX rats treated with alendronate sodium.

**Figure 2:**
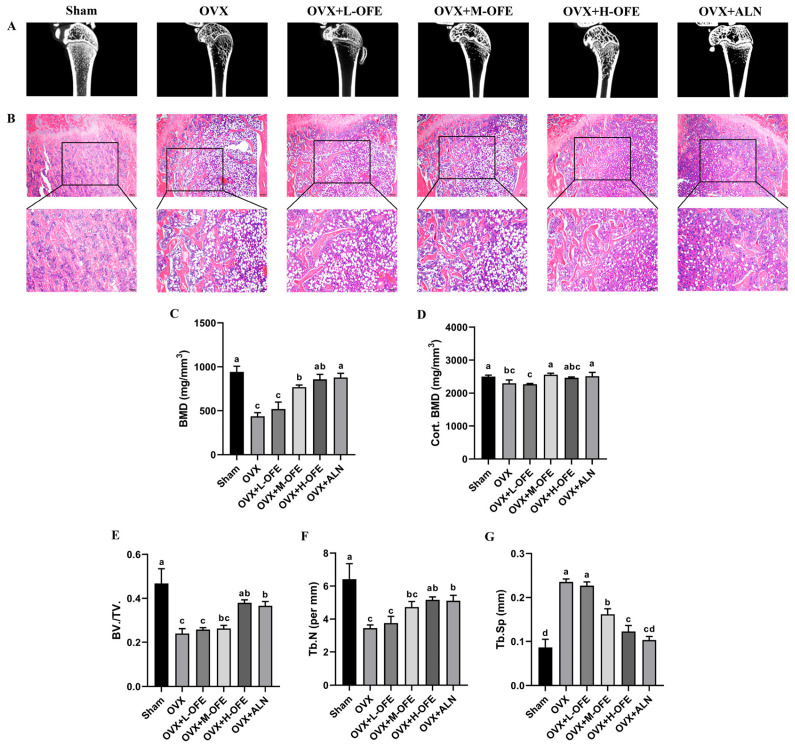
Effects of onion flavonoid extract (OFE) on bone microstructure in ovariectomized (OVX) rats. (**A**) 2D longitudinal section of distal femur. (**B**) Hematoxylin and eosin (H&E) staining of distal femur. (**C**) Bone mineral density (BMD) of the trabecular bone. (**D**) Bone mineral density (BMD) of the cortical bone (Cort. BMD). (**E**) Bone volume per total volume (BV./TV.). (**F**) Trabecular number (Th.N). (**G**) Trabecular separation (Th.Sp). The scar bar is 200 µm (*n* = 3). The different letters represent significant differences between different groups (*p* < 0.05); 80 kV voltage, 500 μA current. The region of interest (ROI) for three-dimensional reconstruction was selected in bone marrow cavity 0.2 mm from the growth plate and had a thickness of 31 μm. OVX+L-OFE: OVX rats treated with low-dose OFE, OVX+M-OFE: OVX rats treated with middle-dose OFE, OVX+H-OFE: OVX rats treated with high-dose OFE, OVX+ALN: OVX rats treated with alendronate sodium.

The design of the animal experimental study is shown in Figure 1. The body weight is shown in Figure S3. To determine the anti-osteoporosis effects of OFE treatment, bone micro-architecture was analyzed by micro-CT. As is shown in Figure 2, it was calculated that BMD declined significantly in ovariectomized (OVX) groups compared with the Sham group (*p* < 0.05), and the net structure of the trabecular was destroyed. Fortunately, this situation was reversed with the oral administration of OFE in a dose-dependent manner. BMD (Figure 2C) in both OVX rats treated with the middle-dose OFE (OVX+M-OFE) group and OVX rats treated with the high-dose OFE (OVX+H-OFE) group was significantly enhanced compared to the OVX group (*p* < 0.05). And trabecular separation (Th.Sp) (Figure 2G) decreased significantly compared with the OVX group (*p* < 0.05). Fortunately, bone volume per total volume (BV./TV.) (Figure 2E) and trabecular number (Th.N) (Figure 2F) were significantly enhanced in the OVX+H-OFE group (*p* < 0.05), while no significant difference was observed between the OVX rats treated with alendronate sodium (OVX+ALN) group (*p* > 0.05). The cortical bone mineral density (Cort. BMD) was also detected in Figure 2D. Cort. BMD decreased significantly in the OVX group compared to the Sham group (*p* < 0.05), while after treatment with M-OFE, Cort. BMD was enhanced significantly compared with the OVX group (*p* < 0.05). And Cort. BMD of rats in the OVX+M-OFE group showed no significance with the OVX+ALN group (*p* < 0.05).

### 2.3. Effect of OFE on the Histopathological Analysis of Bone Tissues of OVX Rats

To characterize the efficacy of OFE on the trabecular structure, the histopathology of the femur was performed in OVX rats by hematoxylin–eosin (H&E) staining. As shown in Figure 1B, the femoral trabecular in OVX groups was disordered, and fracture of the bone trabecular decreased significantly in number and increased in spacing with a lot of fat vacuoles compared with those of the Sham group. The fat vacuoles decreased and the structure of the bone trabecular improved significantly after the 8-week oral administration of OFE treatment, especially in the OVX+H-OFE group.

### 2.4. Effect of OFE on Serum Biochemical Markers

**Figure 3:**
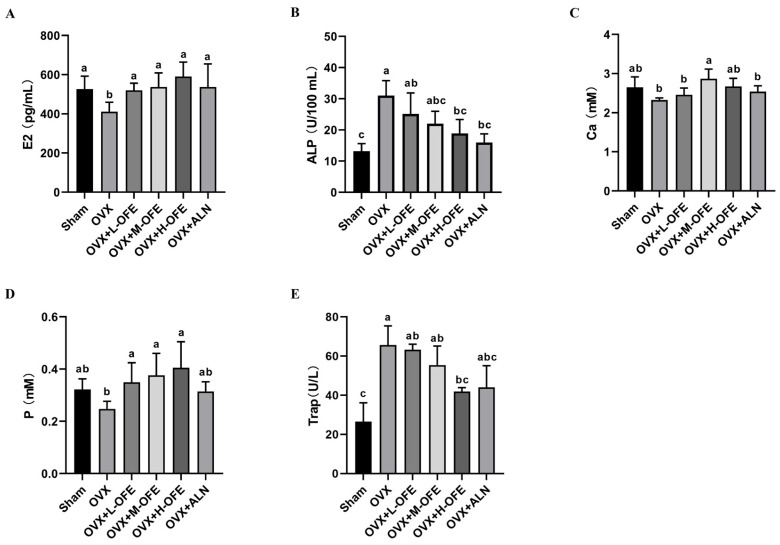
Effects of onion flavonoid extract (OFE) on serum biomarkers in ovariectomized (OVX) rats. (**A**) Estradiol (E2). (**B**) Alkaline phosphatase (ALP). (**C**) Calcium (Ca). (**D**) Phosphorus (P). (**E**) Tartrate-resistant acid phosphatase (TRAP). The different letters represent significant differences between different groups (*p* < 0.05). (*n* = 8) OVX+L-OFE: OVX rats treated with low-dose OFE, OVX+M-OFE: OVX rats treated with middle-dose OFE, OVX+H-OFE: OVX rats treated with high-dose OFE, OVX+ALN: OVX rats treated with alendronate sodium.

It was illustrated that ovariectomy induced a rapid decrease in serum estradiol (E2), serum ALP and Trap increased, and serum calcium and phosphorus were deficient compared with the Sham group (*p* < 0.05, Figure 3). After administration of OFE, the level of E2 (Figure 3A) enhanced significantly in all doses of OFE and alendronate sodium (ALN) compared with the OVX group (*p* < 0.05). The level of Ca (Figure 3C) was enhanced significantly in the M-OFE group compared with the OVX group (*p* < 0.05). And the level of P (Figure 3D) enhanced significantly in all doses of OFE compared with the OVX group (*p* < 0.05). A significant reduction in ALP (Figure 3B) and Trap (Figure 3E) was observed in the OVX+H-OFE group compared with the OVX group as well (*p* < 0.05), while no significant difference was observed between the OVX+ALN group (*p* > 0.05).

### 2.5. OFE Promoted Proliferation and Differentiation of MG-63 Cells

**Figure 4:**
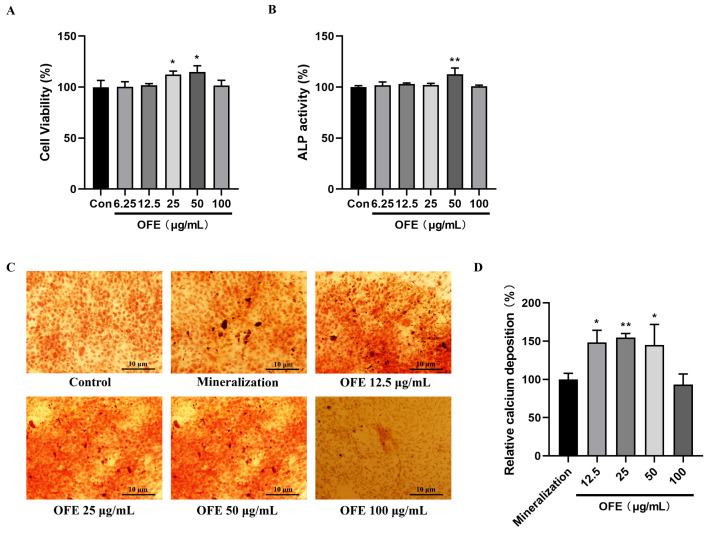
Effects of onion flavonoid extract (OFE) on MG-63 cell proliferation, alkaline phosphatase (ALP) activity, and calcium deposition. (**A**) Methyl thiazolyl tetrazolium (MTT) assay. (**B**) ALP activity. (**C**) Cells were cultured for 21 days after mineralization induction and stained by alizarin red S (ARS). (**D**) Quantitative analysis of relative calcium deposition. The scar bar is 10 µm. Data shown are the mean ± SD of three independent experiments. (*n* = 3) * *p* < 0.05, ** *p* < 0.01 compared with mineralization group. Con: control group. Mineralization: complete DMEM with 50 μg/mL ascorbic acid and 10 mM β-glycerophosphate.

Osteoblasts undergo osteoblast proliferation, extracellular matrix maturation, extracellular matrix mineralization, and osteoblast apoptosis during bone formation. Initially, to ascertain the effects of OFE on the proliferation of MG-63 cells, a methyl thiazolyl tetrazolium (MTT) assay was conducted. MG-63 cells were exposed to various concentrations of OFE for 24 h. As shown in Figure 4A, MG-63 cell proliferation was significantly promoted with OFE at concentrations of 25 and 50 μg/mL compared with the Con group (*p* < 0.01).

The differentiation of osteoblasts occurred later than the proliferation. ALP is a crucial enzyme during the early differentiation period of osteoblasts. Cells were treated with OFE for 4 days, and ALP activity increased significantly at 50 μg/mL OFE (Figure 4B) compared with the Con group (*p* < 0.01). The results showed that OFE promoted the initial stage of osteoblast differentiation by increasing ALP activity.

Calcium deposit is the mean factor of bone matrix mineralization. After induction by a complete medium containing ascorbic acid and β-glycerophosphate, MG-63 cells of both the mineralization group and treatment group (12.5, 25, 50, and 100 μg/mL) formed opaque nodules (Figure 4C), and opaque nodules were formed more in the mineralization groups than were in the control group. Quantitative analysis indicated that the combination of ARS and calcium was increased significantly after MG-63 cells were treated with OFE (12.5, 25, 50 μg/mL) compared with the mineralization group (*p* < 0.05), and as a result, the formation of mineralized nodules was promoted (Figure 4D).

### 2.6. Effect of OFE on OPG/RANKL Pathway

**Figure 5:**
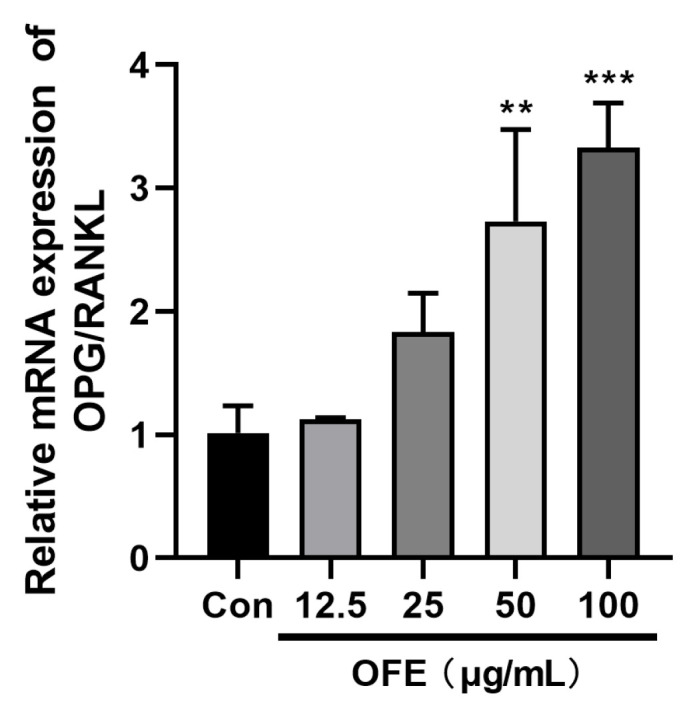
Effects of onion flavonoid extract (OFE) on mRNA expression of osteoprotegerin (OPG)/receptor activator of nuclear factor-kappa B ligand (RANKL) in MG-63 cells. Data shown are the mean ± SD of three independent experiments. (*n* = 3) ** *p* < 0.01, *** *p* < 0.001 compared with the control. Con: control group.

OPG/RANKL is key for bone homeostasis regulation. Therefore, the expression of OPG/RANKL at the mRNA level was explored. As shown in Figure 5, after treatment with OFE, the expression of OPG/RANKL at the mRNA level was significantly upregulated compared with the Con group (*p* < 0.01).

### 2.7. OFE Inhibited the Differentiation of Osteoclasts without Cytotoxicity

**Figure 6:**
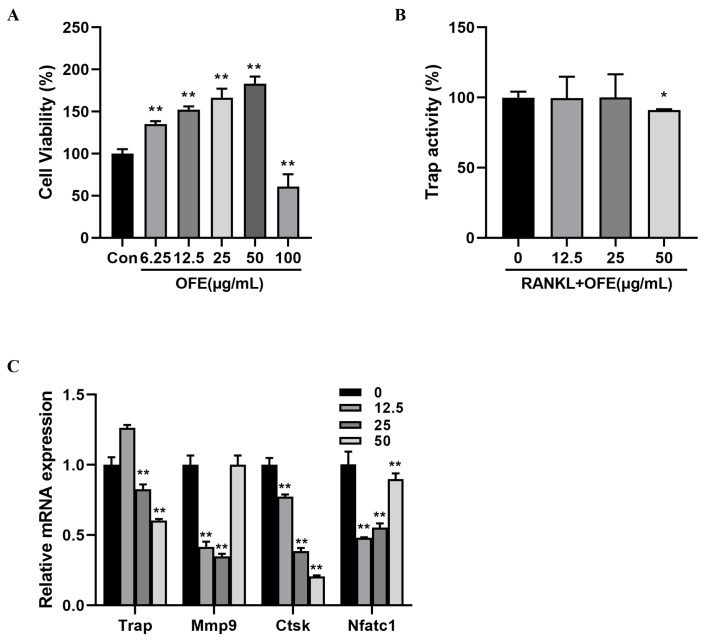
Effects of onion flavonoid extract (OFE) on tartrate-resistant acid phosphatase (TRAP) activity and mRNA expression of osteoclast markers. (**A**) Effects of OFE on RAW 264.7 cell viability. (**B**) TRAP activity in RAW 264.7 cells co-cultured with receptor activator of nuclear factor-kappa B ligand (RANKL) and different concentrations of OFE. (**C**) mRNA expression in RAW 264.7 cells co-cultured with RANKL and different concentrations of OFE. Data shown are the mean ± SD of three independent experiments. (*n* = 3) * *p* < 0.05, ** *p* < 0.01 compared with the control. Con: control group. Mmp9: matrix metalloproteinase 9, Ctsk: cathepsin K, Nfatc1: nuclear factor of activated T cells 1.

To evaluate whether the inhibitory effect of OFE on osteoclastogenesis was attributed to its potential toxicity, RAW 264.7 cell viability was detected using an MTT assay treated with OFE at different concentrations in the absence of RANKL. As expected, the concentration of OFE below 50 μg/mL showed non-toxic effects on cell viability (Figure 6A), indicating that OFE suppressed osteoclastogenesis induced by RANKL.

RANKL-induced osteoclastogenesis from RAW 264.7 cells is a standard of osteoclast differentiation in vitro. TRAP is an essential biomarker for bone resorption, and its activity was explored during the period of osteoclastogenesis. RAW 264.7 cells were co-cultured by RANKL and OFE to determine the effect on osteoclastogenesis. As Figure 6B illustrates, TRAP activity significantly decreased with 50 μg/mL OFE compared with the Con group (*p* < 0.05).

### 2.8. Effect of OFE on Expression of Osteoclast-Specific mRNA

mRNA expression levels of several osteoclast biomarkers were assessed, such as Trap, cathepsin K (Ctsk), and matrix metalloproteinase 9 (Mmp9), in addition to osteoclast-specific nuclear factor of activated T cells 1 (Nfatc-1). During differentiation, mRNA expression levels of Trap, Ctsk, Mmp-9, and Nfatc-1 in cells treated with 50 and 25 μg/mL OFE decreased significantly compared to the Con group (*p* < 0.05) (Figure 6C).

## 3. Discussion

With the aging population, osteoporosis, an age-related metabolic bone disorder, predominantly affects elderly individuals and postmenopausal women. In fact, the incidence of osteoporosis in postmenopausal women can reach as high as 40% [21]. The deficiency of estrogen is widely recognized as a pivotal factor in the pathogenesis of osteoporosis [22]. Consequently, clinical treatment options for postmenopausal osteoporosis encompass bisphosphonates (such as alendronate, risedronate, and ibandronate), denosumab, raloxifene, and menopausal hormone therapy [9]. However, clinical drugs exhibit significant side effects such as upper gastrointestinal tract problems [23], osteonecrosis of the jaw [24], and oculopathy [25]. Therefore, it is imperative to identify an alternative to traditional medicine with a more modest role and minimal side effects. Onion, a vegetable rich in medicinal ingredients with substantial practical potential, particularly flavonoids, demonstrates a beneficial effect on bone health in this study.

After an ovariectomy, a marked decline in estrogen levels was observed in rats, which is a commonly used in vivo model for osteoporosis [26]. Subsequently, osteoclast activity increased while the compensatory enhancement of osteoblast function occurred, resulting in a highly dynamic bone state. Consequently, compared to the Sham group, the OVX group exhibited elevated levels of ALP and Trap in serum, along with decreased Ca and P levels. Additionally, the trabecular bone structure was compromised. Fortunately, bone mineral density was improved significantly by supplementation with OFE when compared to the OVX group (Figure 2), leading to enhanced bone microstructure parameters such as BV./TV., Th.N, and Th.Sp. Furthermore, a significant recovery of serum biomarker levels was observed (Figure 3). Surprisingly, the performance of the OVX+H-OFE group matched that of the OVX+ALN group, a commonly used bisphosphonate drug for treating osteoporosis.

The anti-osteoporosis activity of the flavonoids has already been reported previously. Quercetin has been shown to promote osteoblast differentiation in MC3T3-E1 cells and inhibit osteoclastogenesis in RAW 264.7 cells, indicating a positive impact on bone metabolism [27]. In addition, isoquercitrin and quercetin-3-O-β-d-glucopyranoside, which are also found in onion, promoted the osteogenic differentiation of osteoblasts and BMSCs via the RUNX2 or BMP pathways [28]. Nevertheless, it was reported that a water solution of onion powder benefited the bone by inhibiting osteoclastogenesis [29], while it did not play a role in the activity of osteoblasts, which contradicts our results. This discrepancy may be attributed to variations in active ingredients resulting from different extraction methods or concentrations. Therefore, further investigation is required to identify the specific active components of these flavonoids in onion-derived extracts.

The MG-63 cell line, derived from human osteosarcoma, exhibits rapid cellular proliferation and demonstrates a response pattern similar to that of normal human osteoblast cells, rendering it an ideal model for studying osteoblast cellular behavior. So, it was used as osteoblast in our study [30,31]. It was proven that OFE exerted anti-osteoporosis abilities by stimulating MG-63 cell proliferation and differentiation and inhibiting osteoclastogenesis via the OPG/RANKL signaling pathway. Osteoblasts mediate bone formation by proliferation, differentiation, and matrix mineralization. The results of the MTT assay indicated that the proliferation of osteoblasts could be promoted by OFE (Figure 4A). In general, osteoblasts, proliferating late, begin to differentiate, and the matrix maturation stage is involved in osteoblast differentiation. During the process of maturation, ALP, a biomarker of early differentiation, increases. Meanwhile, osteoblasts perform matrix mineralization during the matrix maturation stage [32]. In the present study, ALP activity in osteoblasts was significantly increased (Figure 3B), and calcium deposition and matrix mineralization were promoted by OFE (Figure 3C), which are key functions of the mature osteoblasts.

The OPG/RANKL/RANK signaling pathway plays a dominant role in interactions between osteoblasts and osteoclasts. Osteoclast activity is close to osteoblast activity via the production of OPG and RANKL. Normally, RANKL binds to its receptor, RANK, on the precursor osteoclasts and osteoclasts to activate the function of osteoclasts. Nevertheless, OPG, secreted by osteoblasts, binds to RANKL competitively as a decoy receptor [33]. As such, the OPG/RANKL ratio indicates the state of bone resorption. It was shown that an increase in the OPG/RANKL ratio weakens bone resorption in turn [34]. In our study, the mRNA expression of the OPG/RANKL ratio was raised after treatment with OFE (Figure 5). This implied that OFE regulated the activity of osteoblasts via the OPG/RANKL signaling pathway. Thus, the molecular triad, OPG/RANKL, serves as a crucial target for preventing osteoporosis [35]. Of course, the OPG/RANKL signaling pathway is regulated by several other pathways, and the mechanism of OFE still needs further exploration.

Osteoclasts are the only recognized, multinucleated cells responsible for bone resorption and are derived from mononuclear precursor cells of the monocyte–macrophage lineage [36]. The interaction between RANKL secreted by osteoblasts and RANK expressed on osteoclasts can facilitate the differentiation and maturation of osteoclasts [37]. The RAW 264.7 cell line induced by RANKL is widely used as a model for studying osteoclast differentiation [6,38,39]. The differentiation of osteoclast precursors into osteoclasts results from the binding of RANKL, secreted by osteoblasts, to its receptor. This increases the expression of the transcription factor NFATc1, which plays an essential role in the activation of osteoclasts and bone resorption [40] and is a pivotal gene during osteoclastogenesis. Furthermore, NFATc1 can generate the mRNA expression of crucial enzymes, such as Ctsk, MMP-9, and TRAP. These enzymes are involved in the degradation of the bone matrix and bone resorption, reflecting the activity of osteoclasts. TRAP is an osteoclast-specific enzyme, marking the state of bone resorption and the quantification of osteoclast numbers [41]. In addition, Ctsk was involved in the digestion of proteinaceous matrix [42], especially breaking down type 1 collagen, which is the main composition of bone matrix. Excessive osteoclast activity and osteoporosis go hand in hand. In this study, the mRNA expression of NFATc1 was down-regulated by OFE (Figure 6). Thereby, OFE inhibited TRAP activity, and meanwhile, the expression of TRAP was down-regulated. Additionally, the expression of Ctsk was down-regulated after the treatment with OFE. Taken together, osteoclast differentiation and osteoclast activity could be inhibited/decreased to block bone resorption by OFE.

## 4. Materials and Methods

### 4.1. Materials and Reagents

Red onions (*Allium cepa* L.) were purchased at a local market in Dalian, China. Rutin (≥98% purity) was purchased from Sichuan Weikeqi Biological Technology (Chengdu, China). The pure water was prepared by Direct-Q 8UV-R (Millipore, St. Louis, MA, USA). All other reagents in the experiments were purchased from Damao chemical reagent factory (Tianjin, China).

### 4.2. Preparation of Allium cepa L. Extract

The red onions were cleaned after the tunics were removed. The bulbs were hot-air-dried (80 °C) for 12 h, and then pulverized. Then, 1 g onion powder was suspended in 15 mL of aqueous ethanol (80%, *v*/*v*) and then extracted in an ultrasonic water bath for 5 min at 25 °C. After being centrifuged at 5000× *g* 5 min, the supernatants were collected. The process was repeated three times, and the supernatants were combined and concentrated under vacuum conditions. Dry OFE powder was stored at −20 °C before use.

Rutin was used as a reference substance and AlCl_3_ as the color-developing agent. The optical density (OD) value at 415 nm was measured colorimetrically, and the total flavonoids were calculated [43]. Colorimetric determination was performed three times.

HPLC-OrbitrapQ-MS was also analyzed. HPLC-OrbitrapQ-MS analysis was performed on a Thermo Fisher Q Exactive OrbitrapQ high-resolution mass spectrometer (Thermo Fisher Scientific, Waltham, MA, USA) equipped with a high-performance liquid chromatography (HPLC) system through an electrospray ionization (ESI) interface. HPLC chromatographic separation was achieved on a ZORBAX SB-C18 (4.6 × 150 mm, 5 μm) column provided by Agilent Technologies (Santa Clara, CA, USA). The mobile phase consisted of formic acid in water (0.1%, *v*/*v*) (A) and acetonitrile (B). The gradient profile was set out as follows: 0–10 min, 10–50% B; 10–13.5 min, 50% B; 14.0–18.0 min, 50–10% B. The flow rate was 1 mL/min. The extracted sample was prepared by diluting in 80% ethanol and filtering through 0.45 μm filter. The temperature of the column was 30 °C. MS and MS/MS data were obtained under negative ion mode during mass spectrum. The mass spectrum conditions were as follows: electron spray ionization (ESI): spray voltage, 400 V; sheath gas, N2; sheath temperature, 400 °C; collision gas, Ar; scan range, *m*/*z* 120–1000.

### 4.3. Animal Experimental Study

#### 4.3.1. Animals and Administration

Forty-eight specific-pathogen-free (SPF) female Sprague Dawley (SD) rats (12 weeks, 250–270 g) were bought from Liaoning Changsheng Biotechnology (Liaoning, China). Rats were housed under standard laboratory conditions (temperature 23 ± 2 °C, humidity 55% ± 5, and 12 h light–dark cycle). All rats drank water and ate food freely. During the experiment, four rats were raised in each cage with a cage size of 545 × 395 × 200 mm. All the animal experiments were conducted with the approval of the Ethics Committee of Dalian University of Technology, China (No. DUTSBE230621-03). All animal experiments in the study were carried out in accordance with the European Community guidelines (Directive 2010/63/EU).

#### 4.3.2. Administration of Animals

After acclimatization for 7 days, rats received either bilateral fat removal of tissue alone (Sham group, *n* = 8) or bilateral ovariectomy (OVX group, *n* = 40). Carbon dioxide was used for anesthesia during the surgery. Meloxicam was used for pain medication for 3 days. Significant bone loss after ovariectomy occurs in the proximal tibial metaphysis after 14 days, in the lumbar vertebral body after 60 days, and in the femoral neck after 30 days [44]. Plasma levels of estradiol in the rats fell rapidly within 5 days after OVX and remained at detectable levels thereafter [26]. After a 6-week recovery period, all the OVX rats were randomly divided into 5 groups: OVX group (*n* = 8), OVX+L-OFE group (*n* = 8, low dose of OFE, 25 mg/kg), OVX+M-OFE group (*n* = 8, middle dose of OFE, 50 mg/kg), OVX+H-OFE group (*n* = 8, high dose of OFE, 75 mg/kg), and OVX+ALN group (*n* = 8, alendronate sodium 1 mg/kg) as the positive control group for 8 weeks. All groups were administered via oral gavage. The rats in Sham group and in OVX group were administered normal saline in the equivalent volume. Body weight was recorded every week. At the end of the 8-week treatment, the rats were euthanized with carbon dioxide. Blood sample was collected from the retro-orbital vein and centrifuged at 1000× *g* for 15 min at 4 °C to separate serum, and all serum samples were kept at −80 °C. The femurs were isolated as quickly as possible, and tissue was rinsed with phosphate buffer solution (PBS) and fixed in 4% neutral formaldehyde (pH 7.0) for further experiments. Based on previous studies, 50 mg/kg OFE was selected as the medium dose [45,46,47,48], with a half-dose of the medium dose (25 mg/kg) and a 1.5-fold increase in the high dose (75 mg/kg) [49]. And, based on the human dose of alendronate of 10 mg/day, a dose of 1 mg/kg/day was selected for rats.

#### 4.3.3. Hematoxylin and Eosin (H&E) Staining

The right femurs were isolated and fixed in 4% neutral formaldehyde (pH 7.0) for 48 h; then, femurs were decalcified in 10% ethylenediaminetetraacetic acid (EDTA) (pH 7.2) at 4 °C for 10 weeks. Then, each bone sample was embedded in paraffin and cut coronally into 4 μm sections. The sections were dewaxed and then stained with hematoxylin and eosin for 5 min, respectively. The sections were dehydrated in a series of ethanol solutions (80%, 90%, 95%, 100%) and xylene and were observed by an IX83 inverted microscope (Olympus Co., Tokyo, Japan)

#### 4.3.4. Micro-CT Analysis

The left femurs were isolated and fixed in 4% neutral formaldehyde (pH 7.0) for 48 h and were scanned using micro-CT (SIEMENS AG, Munich, Germany) at an 80 kV voltage, 500 μA current. X-ray detector pixel size was 34.044 μm. The region of interest (ROI) for three-dimensional reconstruction was selected in bone marrow cavity at 0.2 mm from the growth plate. The selected ROI had a thickness of 31 μm. The trabecular bone volume/tissue volume ratio (BV/TV), trabecular number (Tb. N), trabecular bone separation (Tb. Sp), bone mineral density (BMD), and cortical bone mineral density (Cort. BMD) were analyzed.

#### 4.3.5. Serum Determination

The levels of rat alkaline phosphatase (ALP) (A059-2-2, Nanjing Jiancheng Bioengineering Institute, Nanjing, China), Ca (C004-2-1, Nanjing Jiancheng Bioengineering Institute, Nanjing, China), P (C006-1-1, Nanjing Jiancheng Bioengineering Institute, Nanjing, China), E2 (E-OSEL-R0001, Elabscience, Wuhan, China), and Trap (E-OSEL-R0001, Elabscience, Wuhan, China) in serum were determined by assay kits following the instructions. Absorbance value was measured using a microplate reader (Thermo Fisher Scientific, Waltham, MA, USA).

### 4.4. In Vitro Study

#### 4.4.1. Cell Culture

Both human osteoblast-like cell lines MG-63 and mouse leukemia cells of monocyte–macrophage RAW 264.7 were provided by Chinese Academy of Sciences Cell Bank (Shanghai, China). Cells were cultured in a 37 °C incubator with 5% CO_2_ in Dulbecco modified Eagle’s medium (DMEM, E600008, BBI LIFE SCIENCES, Shanghai, China) supplemented with 10% fetal bovine serum (FBS) and antibiotics (100 IU penicillin/mL and 100 μg streptomycin/mL, E-OSEL-R0001, BBI LIFE SCIENCES, Shanghai, China). MG-63 cells were treated with 6.25, 12.5, 25, 50, or 100 μg/mL OFE for 24 h. RAW 264.7 cells were co-treated with 50 ng/mL RANKL + 12.5, 25, or 50 μg/mL OFE for 6 days.

#### 4.4.2. MTT Assay

The proliferative activity of OFE on MG-63 cells was determined by 3-(4, 5-Dimethyl-2-thiazolyl)-2, 5-diphenyl-2H-tetrazolium bromide (MTT, D274386, Aladdin, Shanghai, China) assay. Stock solutions of OFE were prepared in dimethyl sulfoxide (DMSO, cell culture grade, Solarbio, Beijing, China). Vehicle control groups with 0.1% DMSO were included in the experiments. Stock solutions were diluted by medium to final concentrations of 6.25, 12.5, 25, 50, or 100 μg/mL when they were used. Cells were cultured in 96-well plates with 5 × 10^3^ cells per well for an overnight incubation and were treated with OFE (6.25–100 μg/mL) for 24 h. Then, old medium was removed, and the residual medium with drugs was washed away using sodium phosphate buffer (PBS). Afterwards, 100 μL MTT solution diluted in fresh medium was added per well, and the cells were incubated at 37 °C for another 4 h. After removal of the medium, 100 μL DMSO was added per well to dissolve the formazan crystals. When fully dissolved, the absorbance at 570 nm was measured using a microplate reader (Thermo Fisher Scientific, Waltham, MA, USA), and absorbance at 630 nm was used as reference.

#### 4.4.3. Alkaline Phosphatase (ALP) Activity Assay

For determination of ALP activity, 2 × 10^5^ MG-63 cells per well were seeded in 6-well plates for an overnight incubation and were treated with OFE (6.25–100 μg/mL) for 4d. Total protein was extracted using cell lysates (P0013J, Beyotime Biotechnology, Beijing, China). ALP activity assay was determined by an ALP activity assay kit (A059-2-2, Nanjing Jiancheng Bioengineering Institute, Nanjing, China) performed following the instructions. Absorbance value was measured using a microplate reader (Thermo Fisher Scientific, Waltham, MA, USA).

#### 4.4.4. Alizarin Red S (ARS) Staining

Effect of OFE on mineralization was analyzed by ARS (G1452, Solarbio, Beijing, China) staining. A total of 1 × 10^5^ MG-63 cells per well were seeded in 24-well plates for overnight incubation. After attachment, old medium was replaced by mineralization-induced medium (complete DMEM with 50 μg/mL ascorbic acid (A610021, BBI LIFE SCIENCES, Shanghai, China) and 10 mM β-glycerolphosphate (A500486, BBI LIFE SCIENCES, Shanghai, China)) for the mineralization group and administration group. Cells were treated with 12.5–100 μg/mL OFE for 21 days, and the medium was changed every second day. At the end of culture, cells were rinsed three times with PBS and fixed in 95% ethanol for 20 min at room temperature. After fixation, cells were processed for staining with 1% ARS solution for 30 min at 37 °C. Unbound dye was rinsed with PBS, and the staining was photographed using inverted microscope (IX83, Olympus, Tokyo, Japan).

For quantification of calcium deposition, 500 μL of 10% (*w*/*v*) cetylpyridinium chloride (CPC, A600106, BBI LIFE SCIENCES, Shanghai, China) was added to each well, and plates were incubated at 37 °C for dissolving bound ARS. The absorbance at 550 nm was measured using a microplate reader (Thermo Fisher Scientific, Waltham, MA, USA).

#### 4.4.5. TRAP Activity

RAW 264.7 cells, which can be induced into osteoclasts by RANKL in vitro, were seeded in 6-well plates at a density of 2 × 10^4^ cells per well. After overnight incubation, old medium was replaced with the fresh medium containing 50 ng/mL RANKL (ab307187, Abcam, Cambridge, UK) and OFE. After inducing for 6 days, total proteins were extracted for determination of TRAP activity. TRAP activity was detected with TRAP assay kit following the instructions (E-OSEL-R0001, Elabscience, Wuhan, China). Absorbance value was measured using a microplate reader (Thermo Fisher Scientific, Waltham, MA, USA).

#### 4.4.6. RNA Extraction and Real-Time Reverse Transcription (RT)-PCR

MG-63 cells were seeded into 6-well plates for overnight incubation and were treated with OFE for 24 h. RAW 264.7 cells were seeded into 6-well plates for overnight incubation and co-treated by RANKL and OFE for 6 days. Total RNA was isolated after treatment with Trizol reagent (15596-026, Invitrogen, Waltham, MA, USA), and the quantity and quality of RNA were determined by Nanodrop (Thermofisher, Waltham, MA, USA). And the integrity of RNA was determined by 1% agarose gel electrophoresis. Then, the same amount of total RNA from each sample was used to transcribe reversely with the RevertAidTM first strand cDNA Synthesis Kit (RR047A, Takara, Japan), according to the manufacturer’s instructions. Real-time RT-PCR was performed (PE 7500, PerkinElmer, Waltham, MA, USA) with an SYBR premix ex Taq II kit (RR820A, Takara, Osaka, Japan) for expression of osteoprotegerin (OPG) and receptor activator of nuclear factor kappa-B ligand (RANKL) in MG-63 cells, and glyceraldehyde-3-phosphate dehydrogenase (GAPDH) was used as an internal control [31,50]. In addition, real-time RT-PCR was performed for expression of tartrate-resistant acid phosphatase (Trap), cathepsin K (CtsK), metalloproteinase-9 (Mmp-9), and nuclear factor of activated T-cells, cytoplasmic 1 (Nfatc-1) in RAW 264.7 cells, and Gapdh was used as an internal control [51,52,53]. The primers used to amplify are provided in Table 1. The amplification conditions were as follows: 95 °C for 30 s followed by 40 cycles of 95 °C for 5 s, and 58 °C for 34 s.

### 4.5. Statistical Analysis

All experiments were performed three times. Data were presented as mean ± standard deviation (SD). SPSS 22.0 software was used for statistical analysis, and the significant difference was determined by one-way analysis of variance (ANOVA). The Tukey test was used for subsequent analysis. *p* < 0.05 was considered statistically significant.

## 5. Conclusions

In conclusion, a significant increase in bone mineral density (BMD) and improvement in bone microstructure in OVX rats was demonstrated by the administration of onion flavonoid extract (OFE), comparable to the effects of alendronate sodium. The proliferation and mineralization of MG-63 cells were effectively promoted by OFE, while the mRNA expression of OPG/RANKL increased significantly. Additionally, RANKL-induced osteoclastogenesis of RAW264.7 cells was inhibited by treatment with OFE. Consequently, these findings highlight the potential therapeutic application of OFE for ameliorating osteoporosis as a plant-food bioactive component.

## Figures and Tables

**Table 1:** Primer sequences used in the RT-PCR analysis.

Target mRNA	Primer Sequence (5′-3′)	Fragment Size (bp)
OPG	F: GAAACGTTTCCTCCAAAGTACC R: CTGTCTGTGTAGTAGTGGTCAG	155
RANKL	F: TTACCTGTATGCCAACATTTGC R: TTTGATGCTGGTTTTAGTGACG	103
GAPDH	F: CAGGAGGCATTGCTGATGAT R: GAAGGCTGGGGCTCATTT	138
Trap	F: CAAGAACTTGCGACCATTGTTA R: ATCCATAGTGAAACCGCAAGTA	191
Ctsk	F: GCTTGGCATCTTTCCAGTTTTA R: CAACACTGCATGGTTCACATTA	83
Mmp-9	F: CAAAGACCTGAAAACCTCCAAC R: GACTGCTTCTCTCCCATCATC	105
Nfatc-1	F: GAGAATCGAGATCACCTCCTAC R: TTGCAGCTAGGAAGTACGTCTT	93
Gapdh	F: GGTTGTCTCCTGCGACTTCA R: TGGTCCAGGGTTTCTTACTCC	183

## Data Availability

Data are contained within the article and Supplementary Materials.

## Supplementary Materials

The following supporting information can be downloaded at: https://www.mdpi.com/article/10.3390/ijms25126754/s1.
