# Supplementary material for: Onion (Allium cepa L.) Flavonoid Extract Ameliorates Osteoporosis in Rats Facilitating Osteoblast Proliferation and Differentiation in MG-63 Cells and Inhibiting RANKL-Induced Osteoclastogenesis in RAW 264.7 Cells
Source: Int J Mol Sci. 2024 Jun 19;25(12):6754. doi: 10.3390/ijms25126754 (PMC11203775; doi:10.3390/ijms25126754)
Supplement: Supplementary file 1 [file ijms-25-06754-s001.zip › ijms-2996186-supplementary.pdf]

Supplementary Materials:

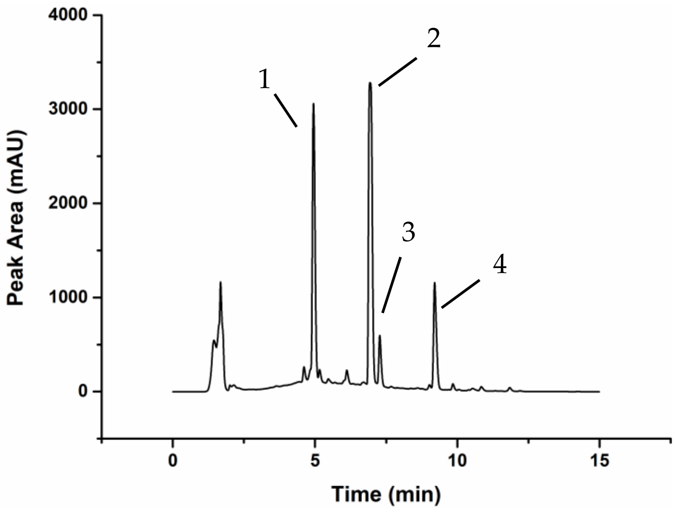

Figure S1 HPLC analysis of OFE.

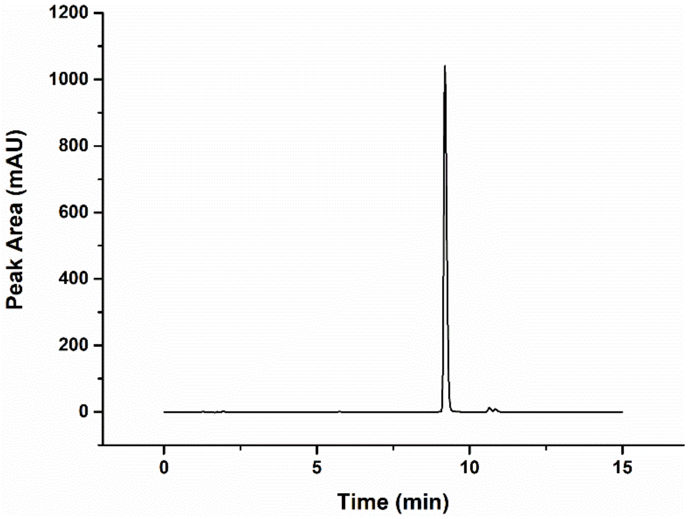

Figure S2 HPLC analysis of quercetin.

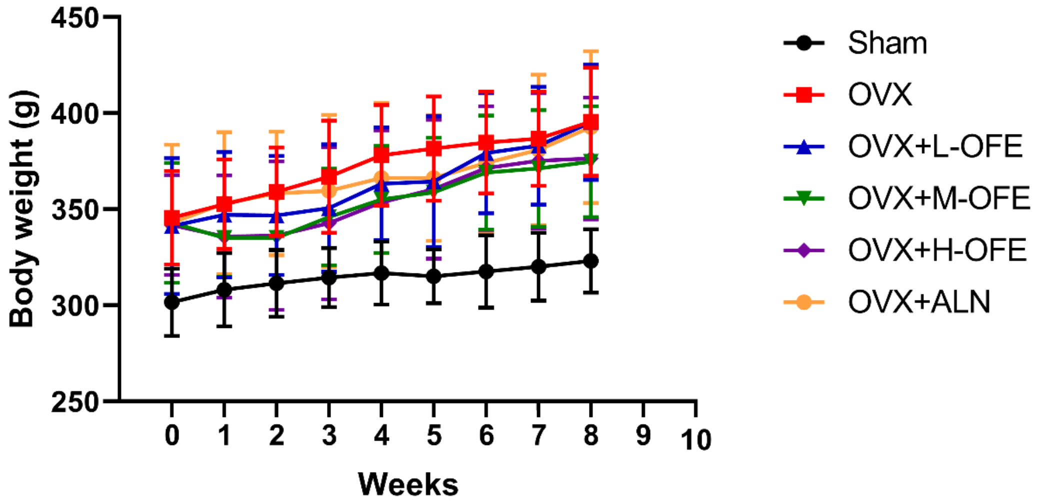

Figure S3 Body weight.

Table S1 HPLC-OrbitrapQ-MS analysis

Retention time (RT)	Formula	$[M-H]^-$	Major fragment ions (MS/MS)
4.9 min	$C_{27}H_{29}O_{17}$	625.1396	463.0863,162
6.9 min	$C_{21}H_{19}O_{12}$	463.0870	301.0342,178.9982,151.0034
7.3 min	$C_{22}H_{21}O_{12}$	477.1030	315.0495
9.2 min	$C_{15}H_{9}O_{7}$	301.0347	151.0033
